# Stress-induced plasticity of GABAergic inhibition

**DOI:** 10.3389/fncel.2014.00157

**Published:** 2014-06-06

**Authors:** Jamie Maguire

**Affiliations:** Department of Neuroscience, Tufts University School of MedicineBoston, MA, USA

**Keywords:** GABA, neurosteroids, stress, KCC2, GABAA receptors, GABAAR, THDOC

## Abstract

GABAergic neurotransmission is highly plastic, undergoing dynamic alterations in response to changes in the environment, such as following both acute and chronic stress. Stress-induced plasticity of GABAergic inhibition is thought to contribute to changes in neuronal excitability associated with stress, which is particularly relevant for stress-related disorders and seizure susceptibility. Here we review the literature demonstrating several mechanisms altering GABAergic inhibition associated with stress, including brain region-specific alterations in GABA_A_ receptor (GABA_A_R) subunit expression, changes in chloride homeostasis, and plasticity at GABAergic synapses. Alterations in the expression of specific GABA_A_R subunits have been documented in multiple brain regions associated with acute or chronic stress. In addition, recent work demonstrates stress-induced alterations in GABAergic inhibition resulting from plasticity in intracellular chloride levels. Acute and chronic stress-induced dephosphorylation and downregulation of the K^+^/Cl^−^ co-transporter, KCC2, has been implicated in compromising GABAergic control of corticotropin-releasing hormone (CRH) neurons necessary for mounting the physiological response to stress. Acute stress also unmasks the capacity for both long-term potentiation and long-term depression, in distinct temporal windows, at GABAergic synapses on parvocellular neuroendocrine cells (PNCs) in the paraventricular nucleus (PVN) of the hypothalamus. This review highlights the complexity in the plasticity of GABAergic neurotransmission associated with stress and the relationship to neuronal excitability, including alterations in GABA_A_R expression, synaptic plasticity at GABAergic synapses, and changes in chloride homeostasis.

Alterations in neuronal excitability and seizure susceptibility associated with stress have largely been attributed to changes in GABAergic inhibition. Here we review the literature describing stress-induced alterations in neuronal excitability and seizure susceptibility associated with stress and the role of GABAergic neurotransmission. Alterations in the expression of GABA_A_Rs and the functional consequences on GABAergic inhibition following both acute and chronic stress in multiple brain regions are discussed. In addition, recent evidence pointing to synaptic plasticity at GABAergic synapses and alterations in intracellular chloride levels resulting in compromised GABAergic inhibition following stress is also highlighted.

## Stress-induced changes in excitability

Anecdotally, it is widely accepted that stress is a precipitating factor for seizures (for review see Maguire and Salpekar, 2013). Patients with epilepsy frequently self-report that stress exacerbates their seizures (Neugebauer et al., 1994; Frucht et al., 2000; Haut et al., 2003, 2007; Nakken et al., 2005; Sperling et al., 2008) and increased cortisol levels are positively correlated with seizure frequency in patients with epilepsy (Galimberti et al., 2005) (for review see Lai and Trimble, 1997; Maguire and Salpekar, 2013). However, the relationship between stress and seizure susceptibility is complex. Broadly speaking, acute stress is thought to be anticonvulsant; whereas, chronic stress is thought to increase seizure susceptibility. This section will review the evidence in animal models documenting changes in seizure susceptibility associated with both acute and chronic stress.

### Acute stress

Swim stress (Soubrie et al., 1980; Pericic et al., 2000, 2001; Reddy and Rogawski, 2002), acute cold stress (de Lima and Rae, 1991), and acute restraint stress (de Lima and Rae, 1991) have been shown to increase seizure threshold (for review see Joels, 2009). The anticonvulsant actions of acute stress are thought to be mediated by the production of stress-derived neurosteroids (for review see Rogawski and Reddy, 2004). In response to stress, deoxycorticosterone (DOC) is released which can be metabolized into the neuroactive derivative, allotetrahydrodeoxycorticosterone (THDOC) which has been demonstrated to exert anticonvulsant actions. Further, DOC itself has also been shown to exhibit anticonvulsant properties, a process which requires neurosteroidogenesis (Reddy and Rogawski, 2002) (for review see Rogawski and Reddy, 2004). The anticonvulsant actions of stress-derived neurosteroids are thought to be mediated by their actions on GABA_A_Rs (for review see Rogawski and Reddy, 2004), which will be discussed more thoroughly in a later section (Stress, seizure susceptibility, and GABA_A_Rs). In addition, recent studies also demonstrate a role for glucocorticoid receptors in the anticonvulsant action of acute stress on seizure susceptibility (Maggio and Segal, 2012), suggesting the involvement of multiple pathways regulating excitability in response to acute stress.

These data clearly demonstrate the anticonvulsant effects of acute stress, which is contrary to the notion that stress is associated with increased seizure susceptibility and is a trigger for seizures in patients with epilepsy (Minter, 1979) (for review see Maguire and Salpekar, 2013). Although acute stress may have anticonvulsant effects, chronic stress appears to increase seizure susceptibility in animal models, a topic which will be covered in more detail in the following section.

### Chronic stress

Not many controlled studies have been undertaken to elucidate the impact of chronic stress on seizure susceptibility. However, the few studies that have been conducted suggest that chronic social isolation or restraint stress increase susceptibility to seizures induced with bicuculline, picrotoxin, kainic acid, and kindling (Matsumoto et al., 2003; Chadda and Devaud, 2004; Jones et al., 2013). Numerous factors likely mediate the effects of chronic stress on neuronal excitability and seizure susceptibility, including effects on synaptic transmission, adult neurogenesis, and hippocampal remodeling (for review see McEwen, 1999, 2000). The hippocampus is a particularly vulnerable region to the adverse effects of stress, which may mediate the increased neuronal excitability and seizure susceptibility associated with chronic stress (for review see McEwen, 1999, 2000). Furthermore, chronic social isolation stress has been shown to decrease the production of neurosteroids (Serra et al., 2000; Dong et al., 2001), which have anticonvulsant properties (for review see Rogawski and Reddy, 2004), and, thereby, may also influence neuronal excitability. Furthermore, early life stress has also been demonstrated to increase the excitability of principal neurons in the hippocampus and increase seizure susceptibility (for review see Koe et al., 2009), suggesting long-term effects of chronic stress on network excitability.

In addition to stress, there is also a relationship between psychiatric disorders and epilepsy (for review see Jones and O'Brien, 2013). Individuals with psychiatric disorders have a greater risk for developing epilepsy (Forsgren and Nystrom, 1990; Hesdorffer et al., 2000, 2006) and is associated with a poorer outcome (Hitiris et al., 2007; Kanner et al., 2009; Petrovski et al., 2010) (for review see Jones and O'Brien, 2013). These studies suggest that chronic anxiety and other stress-related disorders may increase the susceptibility for seizures. Further, this evidence suggests a proconvulsant role for chronic stress and highlights the complex relationship between stress and seizure susceptibility.

## Stress, seizure susceptibility, and GABA_A_Rs

GABA_A_Rs are the primary site of neurosteroid action and likely mediate their anticonvulsant effects (for review see Reddy, 2003). THDOC is produced in response to acute stress, increasing to physiologically-relevant levels which can act on GABA_A_Rs (Purdy et al., 1991; Barbaccia et al., 1996a). Neurosteroids act preferentially at δ-containing GABA_A_Rs (Wohlfarth et al., 2002), but at higher concentrations can also act on different GABA_A_R subtypes (Stell et al., 2003; Belelli et al., 2009). In addition to the direct, positive allosteric modulation of GABA_A_Rs, neurosteroids can also alter the expression of GABA_A_Rs (for review see Maguire and Mody, 2009; Mody and Maguire, 2011). In fact, alterations in the expression of GABA_A_Rs associated with both acute and chronic stress may underlie changes in neuronal excitability and seizure susceptibility which will be discussed in more detail below.

## Stress-induced alterations in gabaergic inhibition

Many studies examining the impact of stress on GABAergic signaling have relied on the expression of GABA synthesizing enzymes and the binding of GABA ligands. Fewer follow up studies have focused on changes in specific GABA_A_R subtypes in specific brain regions and the impact on GABAergic inhibition. Many of these studies have focused on stress-induced changes in GABA in the hippocampus in relation to changes in neuronal excitability (for review see Joels, 2009); however, recent interest has also focused on the role of GABA within the stress neurocircuitry (for review see Gunn et al., 2011), particularly the PVN of the hypothalamus.

### Acute stress

In addition to the direct modulatory effects of acute stress-derived neurosteroids on GABAergic inhibition, acute stress has also been proposed to alter GABAergic inhibition via changes in GABA synthesis, release, and the expression of specific GABA_A_R subunits (for review see Maguire and Mody, 2009; Mody and Maguire, 2011). Changes in GABA synthesis following acute stress have been suggested from alterations in the expression of GABA (Yoneda et al., 1983; Otero Losada, 1988; Acosta et al., 1993) as well as glutamic acid decarboxylase (GAD) (Yoneda et al., 1983; Otero Losada, 1988; Maroulakou and Stylianopoulou, 1991; Acosta et al., 1993; Bowers et al., 1998), the enzyme responsible for the synthesis of GABA (Table 1). GABA levels are decreased in the striatum following acute cold stress (Acosta et al., 1993) and decreased in the olfactory bulb following acute immobilization stress (Otero Losada, 1988). In contrast, GABA levels are increased in the striatum and hypothalamus following acute immobilization stress (Yoneda et al., 1983). GAD expression has been shown to be increased in numerous brain regions following acute thermal stress (Maroulakou and Stylianopoulou, 1991) and acute immobilization/restraint stress (Yoneda et al., 1983; Bowers et al., 1998).

**Table 1:** **Acute stress-induced changes related to GABA**.

	**Stressor**	**Change, direction**	**Brain region**	**Citation**
GABA	Cold stress	Decreased	Corpus striatum	Acosta et al., 1993
	Immobilization stress	Increased	Striatum, hypothalamus	Yoneda et al., 1983
		Decreased	Olfactory bulb	Otero Losada, 1988
		No change	Frontal cortex, hippocampus, medio-basal hypothalamus	Otero Losada, 1988; Yoneda et al., 1983
GAD activity	Cold stress	Decreased	Olfactory bulb′	Acosta et al., 1993
	Thermal stress	Increased	Hypothalamus, hippocampus, striatum, cerebral cortex	Maroulakou and Stylianopoulou, 1991
	Immobilization stress	Increased	Striatum, hypothalamus	Yoneda et al., 1983
GAD67	Acute restraint	Increased	Arcuate nucleus, dorsomedial hypothalamic nucleus, medial preoptic area, BnST, hippocampus	Bowers et al., 1998
GAD65	Acute restraint	Increased	BnST, hippocampus	Bowers et al., 1998
[3H]GABA	Swim stress	Increased	Forebrain	Skerritt et al., 1981
		No change	Cerebellum, cortex, temporal cortex, caudate/putamen, lateral septum, hippocampus, amygdala	Skerritt et al., 1981; Skilbeck et al., 2008
	Cold stress	Decreased	Frontal cerebral cortex, hypothalamus, olfactory bulb	Acosta et al., 1993
	Immobilization stress	Increased	Corpus striatum	Otero Losada, 1988
		Decreased	Olfactory bulb	Otero Losada, 1988
		No change	Frontal cortex, hippocampus, medio-basal hypothalamus	Otero Losada, 1988
	Foot shock	Decreased	Frontal cortex, caudate, cerebellum	Biggio et al., 1981
[3H]diazepam	Swim stress	No change	Forebrain, cerebellum	Skerritt et al., 1981
[3H]flunitrazepam	Cold water swim	Increased	Cortex	Soubrie et al., 1980
		No change	Cerebellum	Soubrie et al., 1980
	Swim stress	Increased	Cerebral cortex	Motohashi et al., 1993
		No change	Hippocampus, cerebellum	Motohashi et al., 1993
	Acute restraint	No change	Cortex	Chadda and Devaud, 2004
[3H]muscimol	Swim stress	No change	Cerebral cortex, hippocampus, cerebellum	Motohashi et al., 1993
[35S]TBPS	CO2 stress	Increased	Cortex	Barbaccia et al., 1996a,b
	Foot shock	Increased	Cortex	Concas et al., 1988
[3H]Ro-15-1788	Acute defeat stress	Increased	Hypothalamus, cortex, cerebellum	Miller et al., 1987
		No change	Midbrain, hippocampus	Miller et al., 1987
	Foot shock	No change	Cortex, hippocampus, striatum, cerebellum, hypothalamus	Drugan et al., 1985
α1	Acute restraint	Decreased	Hippocampus, prefrontal cortex	Zheng et al., 2007
		No change	Striatum	Zheng et al., 2007
γ2	CO2 stress	Decreased	Hippocampus	Maguire and Mody, 2007
δ	CO2 stress	Increased	Hippocampus	Maguire and Mody, 2007
KCC2	Acute restraint	Decreased	PVN	Sarkar et al., 2011
KCC2 P-Ser940	Acute restraint	Decreased	PVN	Sarkar et al., 2011

There are also changes in the expression of GABA_A_Rs following acute stress, evident from changes in the binding of radio-labeled GABA ligands. The binding of [3H]GABA is increased in the forebrain following acute swim stress (Skerritt et al., 1981) and in the striatum following acute immobilization stress (Otero Losada, 1988). In contrast, [3H]GABA binding is decreased in the cortex, hypothalamus and olfactory bulb following acute cold stress (Acosta et al., 1993) and decreased in the olfactory bulb following acute immobilization stress (Otero Losada, 1988). [3H]flunitrazepam binding is increased in the cortex following swim stress (Soubrie et al., 1980; Motohashi et al., 1993; Chadda and Devaud, 2004) and t-[^35^S]butylbicyclophosphorothionate ([^35^S]TBPS) binding is increased in the cortex following either CO_2_ or foot shock stress (Concas et al., 1988; Barbaccia et al., 1996b). The binding of [3H]Ro-15-1788 is decreased in the hypothalamus, cortex, and cerebellum following acute defeat stress (Miller et al., 1987). However, no changes were observed in [3H]diazepam (Skerritt et al., 1981) or [3H]muscimol binding (Motohashi et al., 1993) (Table 1). These data are summarized in Table 1 and demonstrate the complexity in the plasticity of GABA_A_Rs associated with acute stress (for review see Skilbeck et al., 2010). In addition, changes in specific GABA_A_R subunits have been described following acute stress.

Table 1 summarizes changes brain region-specific changes in the expression of specific GABA_A_R subunits associated with several different models of acute stress. Expression of the GABA_A_R α1 and γ2 subunits are decreased in the hippocampus and prefrontal cortex following acute restraint stress or CO_2_ stress (Maguire and Mody, 2007; Zheng et al., 2007). In contrast, the GABA_A_R δ subunit expression is increased following acute stress in the hippocampus following CO_2_ stress (Maguire and Mody, 2007) (Table 1). Functional alterations in GABAergic inhibition following acute stress have also been documented in the hippocampus. Following acute restraint stress, there is an increase in the frequency of spontaneous inhibitory post-synaptic currents (sIPSCs) in CA1 pyramidal cells (Hu et al., 2010). In addition, there is an increase in the tonic GABAergic inhibition recorded in dentate gyrus granule cells (DGGCs) following acute CO_2_ inhalation stress, consistent with the increased expression of the GABA_A_R δ subunit (Maguire and Mody, 2007). Interestingly, some of the alterations in GABA_A_R subunit expression can be mimicked by treatment with THDOC (Maguire and Mody, 2007), suggesting a role for neurosteroids in stress-induced GABA_A_R plasticity.

Alterations in GABAergic inhibition have also been observed in other brain regions following acute stress. The frequency, but not amplitude, of sIPSCs is increased in pyramidal neurons in the prefrontal cortex following acute stress (inescapable shock). Interestingly, these changes were prevented if the animal was able to exert some control over the stress (escapable shock) (Varela et al., 2012). The frequency of sIPSCs is also increased in the PVN following high frequency stimulation in slices from mice subjected to acute restraint stress (Inoue et al., 2013), which is dependent upon glucocorticoid receptor activation and retrograde opioid signaling (Wamsteeker Cusulin et al., 2013). The increased frequency of sIPSCs in the PVN following acute stress may be due to the increased burst firing of GABAergic interneurons in the peri-PVN area (Shin et al., 2011). These data demonstrate alterations in GABAergic inhibition in several different brain regions following acute stress which may involve both pre- and post-synaptic mechanisms.

### Chronic stress

Alterations in GABAergic inhibition also occur following chronic stress, although these changes appear to be unique from those observed following acute stress. Changes in GABA synthesis following chronic stress have been suggested from alterations in the expression of GABA, GAD65, and GAD67 (Table 2). A reduction in the concentration of GABA was observed in the cortex, hypothalamus, and olfactory bulb following chronic cold stress (Acosta et al., 1993). GAD expression is decreased in the striatum and olfactory bulb following chronic cold stress (Acosta et al., 1993); whereas, GAD65 and GAD67 expression are increased in numerous brain regions following chronic intermittent stress (Bowers et al., 1998) and unchanged following repeated swim stress (Montpied et al., 1993). Changes in the expression of GABA_A_Rs following chronic stress have also been suggested from changes in the binding of radio-labeled GABA ligands. [3H]GABA binding is decreased in the hypothalamus following chronic cold stress (Acosta et al., 1993). The binding of [3H]flunitrazepam is decreased in the frontal cortex following chronic foot shock stress and increased following chronic immobilization stress (Braestrup et al., 1979). No change in [3H]flunitrazepam or [3H]muscimol binding was observed following repeated swim stress (Braestrup et al., 1979; Motohashi et al., 1993). These data are summarized in Table 2. Changes in specific GABA_A_R subunits have also been described following chronic stress.

**Table 2:** **Chronic stress-induced changes related to GABA**.

**Measure**	**Stressor**	**Change/direction**	**Brain region**	**Citation**
GABA	Cold stress	Decreased	Frontal cerebral cortex, hypothalamus, and olfactory bulbs	Acosta et al., 1993
GAD	Cold stress	Decreased	Corpus striatum, olfactory bulb	Acosta et al., 1993
	Repeated swim stress (7 day)	No change	Hippocampus	Montpied et al., 1993
	Repeated swim stress (14 day)	No change	Hippocampus	Montpied et al., 1993
GAD67	Chronic intermittent stress	Increased	Medial preoptic area, BnST, hippocampus	Bowers et al., 1998
GAD65	Chronic intermittent stress	Increased	Anterior hypothalamic area, dorsomedial nucleus, medial preoptic area, suprachiasmatic nucleus, BnST, perifornical nucleus, periparaventricular nucleus	Bowers et al., 1998
[3H]GABA	Cold stress	Decreased	Hypothalamus	Acosta et al., 1993
[3H]flunitrazepam	Repeated swim stress	No change	Cerebral cortex, hippocampus, cerebellum, striatum, occipital cortex	Motohashi et al., 1993; Braestrup et al., 1979
	Chronic foot shock	Decreased	Frontal cortex	Braestrup et al., 1979
	Chronic foot shock	No change	Striatum, occipital cortex	Braestrup et al., 1979
	Chronic immobilization stress	Increased	Frontal cortex	Braestrup et al., 1979
	Chronic immobilization stress	No change	Striatum	Braestrup et al., 1979
[3H]muscimol	Repeated swim stress	No change	Cerebral cortex, hippocampus, cerebellum	Motohashi et al., 1993
α1	Repeated swim stress (7 day)	No change	Hippocampus	Montpied et al., 1993
	Repeated swim stress (14 day)	Decreased	Hippocampus	Montpied et al., 1993
	Chronic unpredictable stress	No change	PVN	Verkuyl et al., 2004
	Social isolation	Decreased	Frontal cortex	Matsumoto et al., 2007
α2	Repeated swim stress (7 day)	No change	Hippocampus	Montpied et al., 1993
	Repeated swim stress (14 day)	No change	Hippocampus	Montpied et al., 1993
	Social isolation	Decreased	Frontal cortex	Matsumoto et al., 2007
α3	Repeated swim stress (7 day)	No change	Hippocampus	Montpied et al., 1993
	Repeated swim stress (14 day)	No change	Hippocampus	Montpied et al., 1993
	Chronic unpredictable stress	No change	PVN	Verkuyl et al., 2004
α4	Chronic unpredictable stress	No change	PVN	Verkuyl et al., 2004
	Social isolation	Increased	Frontal cortex	Matsumoto et al., 2007
		Increased	Hippocampus	Serra et al., 2006
α5	Chronic unpredictable stress	Increased	PVN	Verkuyl et al., 2004
	Social isolation	Increased	Frontal cortex	Matsumoto et al., 2007
β1	Chronic unpredictable stress	No change	PVN	Verkuyl et al., 2004
		Increased	PVN	Cullinan and Wolfe, 2000
β2	Chronic unpredictable stress	No change	PVN	Verkuyl et al., 2004
		Increased	PVN	Cullinan and Wolfe, 2000
		Decreased	Hippocampus	Cullinan and Wolfe, 2000
β3	Chronic unpredictable stress	No change	PVN	Verkuyl et al., 2004
δ	Chronic unpredictable stress	Decreased	PVN	Verkuyl et al., 2004
		Increased	Hippocampus	Serra et al., 2006
γ1	Chronic unpredictable stress	No change	PVN	Verkuyl et al., 2004
γ2	Chronic unpredictable stress	No change	PVN	Verkuyl et al., 2004
γ3	Chronic unpredictable stress	No change	PVN	Verkuyl et al., 2004
π	Chronic unpredictable stress	No change	PVN	Verkuyl et al., 2004
KCC2	Chronic defeat stress	Decreased	PVN	Miller and Maguire, 2014
KCC2 P-Ser940	Chronic defeat stress	Decreased	PVN	Miller and Maguire, 2014

A decrease in the expression of the GABA_A_R α1 subunit has been observed in the hippocampus following repeated swim stress with no change observed in α2 or α3 (Montpied et al., 1993). Similarly, a decrease in the expression of the GABA_A_R β 2 subunit was observed in the hippocampus following chronic unpredictable stress (Cullinan and Wolfe, 2000). An upregulation of α5 and β 1 subunits have been observed in the PVN following chronic unpredictable stress (Cullinan and Wolfe, 2000; Verkuyl et al., 2004); whereas, a decrease in the expression of the δ subunit was observed in the PVN with no change in α1, α3, α4, γ1, γ2, γ3, or π expression (Montpied et al., 1993; Verkuyl et al., 2004). Chronic social isolation stress results in a decrease in α1 and α2 expression and an increase in α4 and α5 expression in the frontal cortex (Matsumoto et al., 2007). Similarly, an increase in α4 and δ subunit expression has been observed in the hippocampus following chronic social isolation stress (Serra et al., 2006). These changes are summarized in Table 2, highlighting the brain region-specific alterations in GABA_A_Rs following chronic stress. Consistent with changes in GABA_A_R subunit expression, functional changes in GABAergic inhibition have been observed following chronic stress. An increase in the frequency of sIPSCs has been observed in CA1 pyramidal neurons following chronic restraint stress (Hu et al., 2010), which is mediated by glucocorticoid receptor activation (Hu et al., 2010). In contrast, a decrease in the frequency of sIPSCs has been observed following chronic mild stress in DGGCs (Holm et al., 2011), which is consistent with a decrease in the expression of the α1/α2 and γ2 subunits (Matsumoto et al., 2007). Consistent with an upregulation of the GABA_A_R α4, α5 and δ subunit (Serra et al., 2006; Matsumoto et al., 2007), an increase in tonic GABAergic inhibition has been measured in DGGCs following chronic stress (Serra et al., 2008; Holm et al., 2011). In the PVN, a decrease in the frequency of sIPSCs has been observed (Verkuyl et al., 2004), which can be mimicked with exogenous corticosterone (Verkuyl et al., 2005) and reversed with adrenalectomy (Verkuyl and Joels, 2003). These data demonstrate the brain region-specific plasticity in GABAergic neurotransmission associated with chronic stress, which may underlie changes in neuronal excitability but also changes in sensitivity to pharmacological compounds.

Impaired GABAergic inhibition following chronic stress may also result from a decrease in neurosteroid synthesis. A decrease in the production of 3α,5α-tetrahydroprogesterone (3α,5α-THP; allopregnanolone) has been observed following chronic stress (Serra et al., 2000; Dong et al., 2001; Pinna et al., 2003; Matsumoto et al., 2007). Decreased levels of neurosteroids may limit the allosteric modulation of GABA_A_Rs as well as potentially contribute to the observed changes in GABA_A_R subunit expression (for review see Maguire and Mody, 2009). The decreased production of endogenous positive modulators of GABA_A_Rs, combined with alterations in GABA_A_R subunit expression and decreased binding of GABA ligands, indicates altered GABAergic signaling in multiple brain regions following chronic stress.

## Stress-induced alterations in chloride homeostasis

Effective GABAergic inhibition requires the maintenance of the chloride gradient, which is accomplished by the K^+^/Cl^−^ co-transporter, KCC2, in the adult brain (Rivera et al., 1999, 2005; Payne et al., 2003). Recent studies have begun to investigate the impact of changes in chloride homeostasis on GABAergic inhibition under both physiological and pathological conditions, including following acute and chronic stress.

### Acute stress

Our lab recently demonstrated dynamic changes in GABAergic inhibition in CRH neurons in the PVN following acute restraint stress. CRH neurons are at the apex of control of the hypothalamic-pituitary-adrenal (HPA) axis, which mediates the body's physiological response to stress. Following acute restraint stress, KCC2 is dephosphorylated at residue Ser 940 and downregulated in the PVN (Sarkar et al., 2011) (Table 1). Although functional deficits in KCC2 transport have not directly been measured following acute stress, shifts in E_GABA_ and compromised GABAergic control of CRH neurons (Hewitt et al., 2009; Sarkar et al., 2011) are thought to result from the dephosphorylation and downregulation of KCC2, leading to excitatory actions of GABA (Sarkar et al., 2011). The changes in KCC2 and GABAergic inhibition following acute stress are unique to these CRH neurons and we believe are part of the signaling cascade required to mount a rapid, all-or-none response to stress.

### Chronic stress

Alterations in KCC2 in the PVN, such as those observed following acute stress, persist following chronic social defeat stress (Miller and Maguire, 2014). The dephosphorylation and downregulation of KCC2 in the PVN is accompanied by stress-induced elevations in corticosterone throughout the chronic social defeat stress paradigm (Miller and Maguire, 2014). These data are consistent with the role of dephosphorylation and downregulation of KCC2 in the PVN in mounting the physiological response to stress. However, deficits in KCC2 transporter function have not been directly measured following chronic stress and future studies are required to determine the significance of these changes on neuronal excitability. Interestingly, the ability of neurosteroids to potentiate GABAergic inhibition and limit the activity of PNCs is reduced following early life stress due to compromised GABAergic inhibition associated with a shift in E_GABA_ in PNCs (Gunn et al., 2013). Thus, it appears that chronic stress impairs the GABAergic control of PNCs, via downregulation of KCC2, which has significant implications for KCC2 as a therapeutic target. Stress-induced changes in chloride plasticity in other brain regions and the impact on neuronal excitability and seizure susceptibility remains to be explored, but are necessary steps given the interest in targeting KCC2 for therapeutics.

## Plasticity in the gabaergic control of the HPA axis

In addition to changes in GABAergic neurotransmission resulting from changes in GABA_A_R subunit expression and/or chloride plasticity, elegant studies have demonstrated stress-induced synaptic plasticity at GABAergic synapses following acute stress. Acute restraint stress has been demonstrated to unmask the capacity for activity-dependent long-term potentiation at GABAergic synapses (LTP_GABA_) on PNCs in the PVN, a process which involves the activation of β-adrenergic receptors and an upregulation of mGluR1 receptors (Inoue et al., 2013). This potentiation of GABAergic inhibition has been proposed to overwhelm the chloride extrusion mechanisms (Inoue and Bains, 2014), accomplished by KCC2, which accounts for the collapse in the chloride gradient following stress (Hewitt et al., 2009; Sarkar et al., 2011). Interestingly, there is temporal specificity in the plasticity of GABAergic inhibition on PNCs. At 90 min post-restraint stress, GABAergic synapses on PNCs exhibit long-term depression (LTD_GABA_), mediated by the actions of glucocorticoids (Wamsteeker Cusulin et al., 2013) which is thought to limit the stress response. These data demonstrate the bidirectional plasticity in GABAergic inhibition on PNCs following acute stress and highlight the importance of synaptic plasticity and the dynamic impact on GABAergic neurotransmission in the regulation of the HPA axis.

## Concluding remarks

Here we review the role of stress in the plasticity of GABAergic inhibition. In addition to changes in GABA_A_R subunit expression, recent evidence demonstrates a greater complexity in the plasticity of GABAergic neurotransmission associated with stress, involving changes in chloride homeostasis and synaptic plasticity at GABAergic synapses. These data suggest that there may be additional targets, other than GABA_A_Rs, for managing the impact of stress on GABAergic neurotransmission which has implications for stress-related disorders and seizure susceptibility.

### Conflict of interest statement

The author declares that the research was conducted in the absence of any commercial or financial relationships that could be construed as a potential conflict of interest.
